# Enhancing mitochondrial pyruvate metabolism ameliorates ischemic reperfusion injury in the heart

**DOI:** 10.1172/jci.insight.180906

**Published:** 2024-07-25

**Authors:** Joseph R. Visker, Ahmad A. Cluntun, Jesse N. Velasco-Silva, David R. Eberhardt, Luis Cedeño-Rosario, Thirupura S. Shankar, Rana Hamouche, Jing Ling, Hyoin Kwak, J. Yanni Hillas, Ian Aist, Eleni Tseliou, Sutip Navankasattusas, Dipayan Chaudhuri, Gregory S. Ducker, Stavros G. Drakos, Jared Rutter

**Affiliations:** 1 Nora Eccles Harrison Cardiovascular Research and Training Institute and; 2 Department of Biochemistry, School of Medicine, University of Utah, Salt Lake City, Utah, USA.; 3 Division of Cardiovascular Medicine, Department of Internal Medicine, School of Medicine, Salt Lake City, Utah, USA.; 4 Department of Biomedical Engineering, School of Medicine, University of Utah, Salt Lake City, Utah, USA.; 5 Howard Hughes Medical Institute, Chevy Chase, Maryland, USA.

**Keywords:** Cardiology, Metabolism, Carbohydrate metabolism, Cardiovascular disease, Mitochondria

## Abstract

The clinical therapy for treating acute myocardial infarction is primary percutaneous coronary intervention (PPCI). PPCI is effective at reperfusing the heart; however, the rapid reintroduction of blood can cause ischemia-reperfusion (I/R). Reperfusion injury is responsible for up to half of the total myocardial damage, but there are no pharmacological interventions to reduce I/R. We previously demonstrated that inhibiting monocarboxylate transporter 4 (MCT4) and redirecting pyruvate toward oxidation can blunt hypertrophy. We hypothesized that this pathway might be important during I/R. Here, we establish that the pyruvate-lactate axis plays a role in determining myocardial salvage following injury. After I/R, the mitochondrial pyruvate carrier (MPC), required for pyruvate oxidation, is upregulated in the surviving myocardium. In cardiomyocytes lacking the MPC, there was increased cell death and less salvage after I/R, which was associated with an upregulation of MCT4. To determine the importance of pyruvate oxidation, we inhibited MCT4 with a small-molecule drug (VB124) at reperfusion. This strategy normalized reactive oxygen species (ROS), mitochondrial membrane potential (ΔΨ), and Ca^2+^, increased pyruvate entry to the TCA cycle, increased oxygen consumption, and improved myocardial salvage and functional outcomes following I/R. Our data suggest normalizing pyruvate-lactate metabolism by inhibiting MCT4 is a promising therapy to mitigate I/R injury.

## Abstract 2

We investigated a pharmacological approach to boost mitochondrial function and reduce the damage after a heart attack, which is a leading cause of death worldwide.

## Graphical abstract

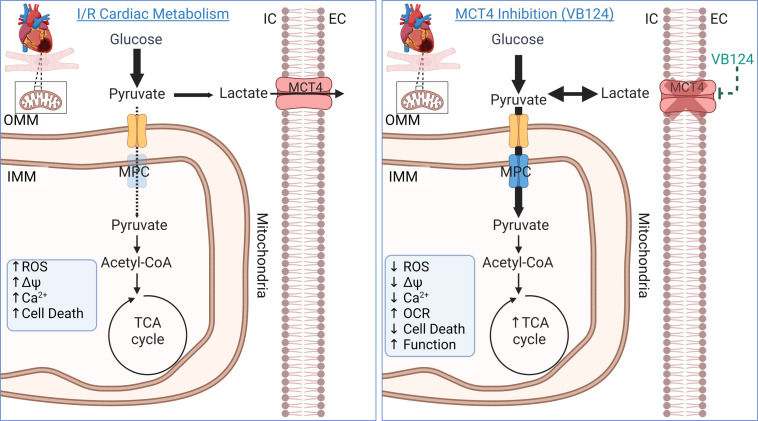

## Introduction

Acute myocardial infarction (AMI) is a major public health problem, a principal cause of heart failure (HF), and a leading cause of mortality worldwide (1–3). The standard of care for patients with AMI is primary percutaneous coronary intervention (PPCI) to reperfuse and restore oxygenated blood flow to the ischemic myocardium (4, 5). Paradoxically, PPCI is accompanied by reperfusion injury, which exacerbates tissue injury and increases cardiomyocyte death resulting in a reduction of salvageable myocardium. It is estimated that reperfusion injury accounts for approximately 50% of the final infarct after an AMI (4, 6). Despite decades of research, no pharmacological interventions have been successfully translated into routine clinical practice to alleviate the detrimental effects of ischemia-reperfusion (I/R) injury (7–9). Therefore, mitigation of myocardial I/R injury remains an unmet need in cardiovascular medicine to prevent the development of chronic HF following ischemic events.

The mechanisms underlying I/R are complex and multifactorial, but data from animal models suggest that a key contributor is mitochondrial dysfunction within the ischemic cardiomyocytes (10–12). Mitochondrial function is critical in cardiomyocytes during I/R injury to maintain cellular energetics, redox, and viability (13). Mitochondrial defects in response to I/R injury, which can lead to mitochondria-mediated apoptosis, include impaired mitochondrial membrane potential (ΔΨ), calcium overload, and oxidative stress (14, 15). This is thought to arise in I/R due to a metabolic imbalance caused by the discontinuous availability of oxygen and nutrients during I/R (16, 17). Understanding the metabolic perturbations associated with I/R could enable the development of new cardioprotective therapies to minimize the damage associated with I/R and prevent subsequent HF.

The mitochondrial pyruvate carrier (MPC) is an inner mitochondrial membrane obligate heterodimeric transporter (composed of MPC1 and MPC2), which facilitates entry of pyruvate into the mitochondrial matrix where it can be converted into acetyl-CoA by the pyruvate dehydrogenase complex to feed the tricarboxylic (TCA) cycle (18). In the relatively hypoxic fetal heart, cardiac energy production is met largely by carbohydrate fuels and glycolytic metabolism. This metabolism also enables biomass production for the growing heart. However, a metabolic transition occurs at birth and the mature myocardium derives approximately 60%–90% of its ATP from fatty acid oxidation (19).

During an ischemic event, the supply of oxygen and nutrients to the myocardium is reduced, which is partially compensated for by an increase in glucose consumption, but results in a build-up of electrons in the form of NADH and succinate and increased production of lactate due to the disruption in aerobic respiration (20, 21). Upon reperfusion of the ischemic myocardium, the sudden influx of oxygen and nutrients elicits a metabolic imbalance that causes a surge of reactive oxygen species (ROS) resulting from rapid succinate oxidation (11, 12). The MPC is upregulated in the periinfarct border zone of I/R injury and may be cardioprotective by contributing to increased energy production, reduced oxidative stress, and the promotion of prosurvival pathways (22). We and others recently showed that adult cardiomyocyte-specific deletion of *Mpc1* caused cardiac hypertrophy and HF (23–25). When pyruvate cannot enter the TCA cycle via the MPC it is converted into lactate by the activity of lactate dehydrogenase (LDH). Lactate is exported from cardiomyocytes by the activity of monocarboxylate transporter 4 (*SLC16A3* or MCT4) (20, 21, 26). We have defined this dynamic interplay between pyruvate and lactate as the pyruvate-lactate metabolic axis (18).

Utilizing both in vitro and in vivo models of I/R, we show that cardiomyocyte-specific deletion of *Mpc1* leads to less myocardial salvage and greater overall injury. Moreover, *Mpc1*-deficient (*Mpc1*^CKO^) hearts exhibited an approximately 20% increase in myocardial necrosis following I/R, accompanied by increased MCT4 expression. Based on these observations, we tested the hypothesis that inhibiting lactate export and redirecting pyruvate carbon through the MPC into mitochondrial oxidation would be protective in models of I/R injury. We employed the small-molecule MCT4 inhibitor VB124 in both in vitro and in vivo models of acute and chronic I/R injury (18). We found that MCT4 inhibition normalized ROS, ΔΨ, Ca^2+^ levels, and pyruvate oxidation, leading to increased myocardial salvage and improved cardiac function. Therefore, building upon an understanding of the metabolic mechanisms surrounding I/R, our data suggest that targeting the pyruvate-lactate metabolic axis with MCT4 inhibition is a promising cardioprotective therapy to ameliorate I/R injury.

## Results

### Murine hearts lacking the MPC have less myocardial salvage and more necrosis following I/R injury.

**Figure 1:**
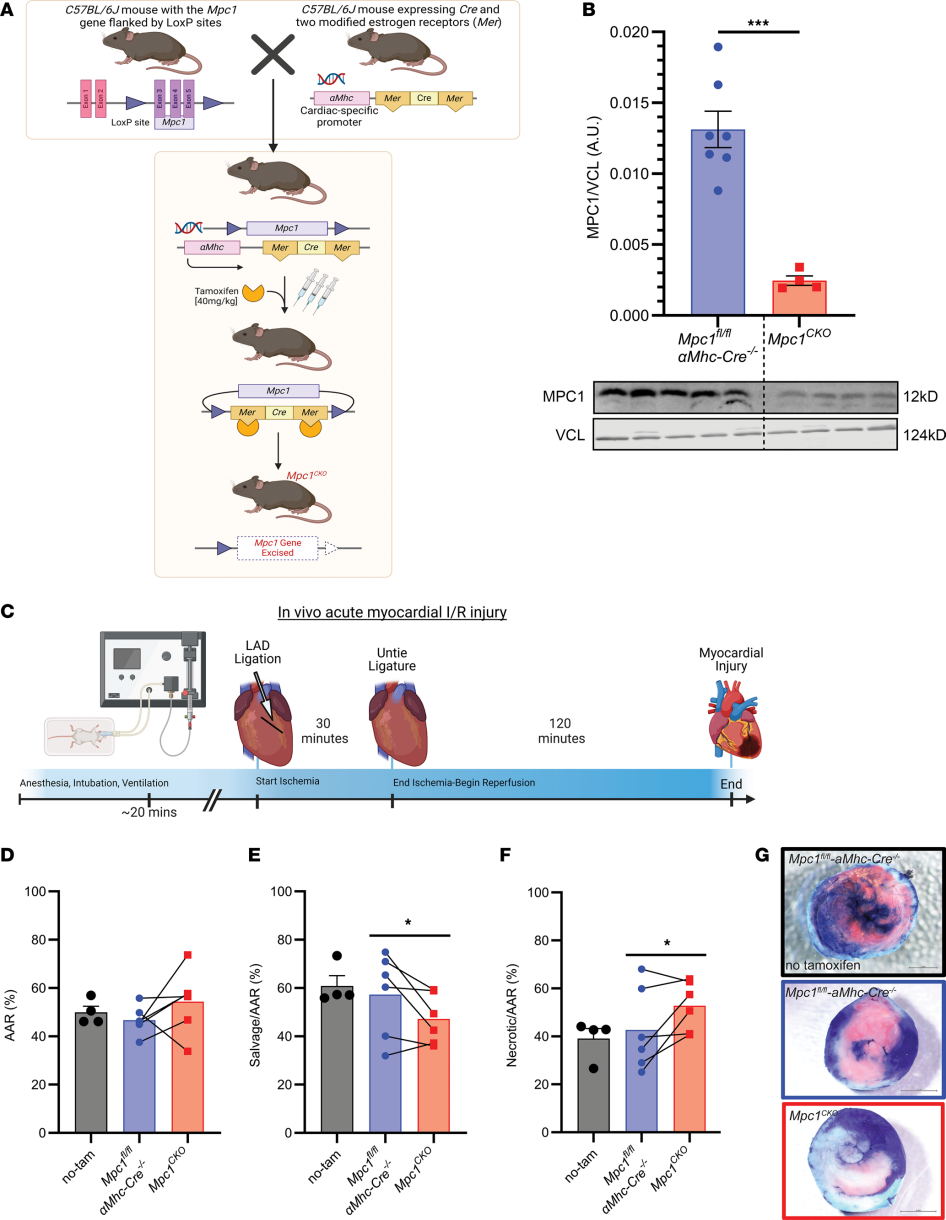
Loss of the MPC in murine hearts results in less myocardial salvage with more necrosis following I/R. (**A**) The *Mpc1* gene locus is targeted in *C57Bl/6J* mice by placing loxP sites in the introns flanking exons 3–5 of the *Mpc1* genomic locus. Cardiomyocyte specificity was engineered by crossing *Mpc1^fl/fl^* mice with an *Mhc* driven tamoxifen inducible Cre. To induce deletion of *Mpc1* (*Mpc1^CKO^*), mice were intraperitoneally injected with tamoxifen for 3 consecutive days (40 mg/kg) at 8 weeks old. (**B**) Western blot showing significant knockdown of MPC1 from whole hearts (2-tailed, Student’s *t* test) in *Mpc1^fl/fl^-*α*mhc-cre^–/–^*(*n* = 7) and *Mpc1^CKO^* (*n* = 4). Samples were run in parallel with one another, at the same time on separate gels blotting for MPC1 and VCL. (**C**) Schematic of in vivo myocardial I/R injury model. (**D**) Following I/R injury, the area at risk (AAR%) was nonsignificant (*P* = 0.13), indicating similar initial ischemic injury between tamoxifen treated *Mpc1^fl/fl^-*α*mhc-cre^–/–^* (46.67±2.45%, *n* = 6) and *Mpc1^CKO^* (54.33±5.42%, *n* = 6). (**E** and **F**) Within the area at risk, myocardial salvage (TTC: pink tissue staining) is significantly reduced (*P* = 0.04) in the *Mpc1^CKO^* (47.21±4.22%, *n* = 6) when compared with their paired tamoxifen treated *Mpc1^fl/fl^-*α*mhc-cre^–/–^* littermates (57.28±7.09%, *n* = 6). (**G**) Representative images of myocardial salvage and necrosis following in vivo I/R injury in nontamoxifen treated *Mpc1^fl/fl^-*α*mhc-cre^–/–^* (no-tam), and tamoxifen treated *Mpc1^fl/fl^-*α*mhc-cre^–/–^*, and *Mpc1^CKO^* mice. Paired *t* test was used for statistical analysis between the tamoxifen treated *Mpc1^fl/fl^-*α*mhc-cre^–/–^* and *Mpc1^CKO^* mice (**D**–**F**). There were no differences seen in the AAR, or myocardial salvage and necrosis between tamoxifen and nontamoxifen-treated (*n* = 4) *Mpc1^fl/fl^-*α*mhc-cre^–/–^* animals (2-tailed, Student’s *t* test). *P < 0.05, ***P < 0.001. Values are represented as mean±SEM. BioRender was used to make panels **A** and **C**.

We used a cardiac-specific, tamoxifen-inducible conditional *Mpc1-*deletion mouse model to investigate the role of MPC1 in the myocardial response to I/R (Figure 1A). Four weeks postinjection (WPI) with tamoxifen, and prior to any observable cardiovascular phenotype, we subjected *Mpc1^CKO^* (tamoxifen treated *Mpc1^fl/fl^-*α*mhc-cre^+/–^)* mice and control mice (tamoxifen treated *Mpc1^fl/fl^-*α*mhc-cre^–/–^*, hereafter referred to as WT) to in vivo myocardial I/R injury (Figure 1B and C) and assessed the extent of myocardial damage. Using the double-staining histological technique of Evans blue and triphenyltetrazolium chloride (TTC) (27–31), we controlled for infarct size and found that the area at risk (AAR) was indistinguishable between WT and *Mpc1^CKO^* hearts, indicating a similar initial ischemic insult (Figure 1D). However, when normalized to the AAR, the myocardial salvage was reduced by 20% in *Mpc1^CKO^* mice compared with their paired WT littermates (Figure 1E–G). Moreover, there were no differences seen in the AAR or myocardial salvage and necrosis between tamoxifen and nontamoxifen treated WT animals (Figure 1G).

### Mpc1^CKO^ mice upregulate several deleterious pathways upon I/R.

**Figure 2:**
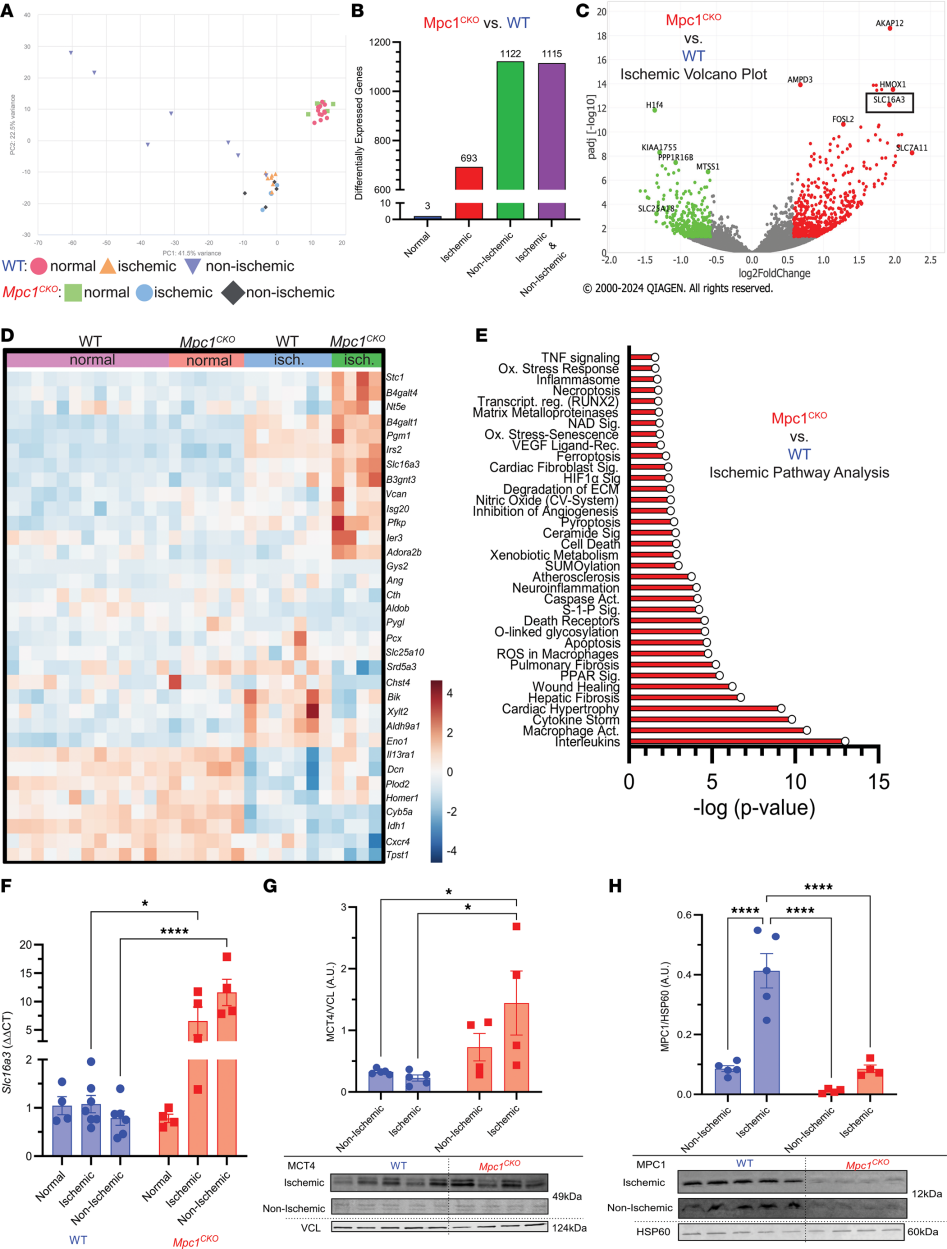
*Mpc1^CKO^* mice upregulate several deleterious pathways upon I/R. (**A**) Principal component analysis plot showing gene expression profiles of WT (normal: *n* = 13, ischemic: *n* = 7, nonischemic: *n* = 7) and *Mpc1^CKO^* (normal: *n* = 6, ischemic: *n* = 4, nonischemic: *n* = 4) data sets. (**B**) Bar graph showing the distribution of significant genes within experimental conditions. (**C**) Volcano plot of genes identified using IPA in the *Mpc1^CKO^* ischemic myocardium, including *Slc16a3* (MCT4, black box). (**D**) Heatmap of genes associated with glycolysis in WT and *Mpc1^CKO^* hearts and their response to I/R injury showing an upregulation of *Slc16a3* in MPC1^CKO^ hearts. (**E**) IPA pathway analysis reveals that within the ischemic tissue of *Mpc1^CKO^* hearts there is an association with deleterious pathways such as interleukin signaling, macrophage activation, cytokine storm activity, and cell death. (**F**) qRT-PCR showing *Slc16a3* expression is normally low in both WT and *Mpc1^CKO^* hearts but upon I/R injury *Slc16a3* gene expression is upregulated in the ischemic and nonischemic samples of only the *Mpc1^CKO^*. (**G** and **H**) The relative protein abundance via Western blotting of MCT4 and MPC1 in the nonischemic and ischemic myocardium reveal an unbalanced pyruvate-lactate metabolic axis following I/R injury. 2-way ANOVAs with a Tukey’s HSD post hoc test were used for statistical analysis between WT (*n* = 5) and *Mpc1^CKO^* (*n* = 4), and ischemic, nonischemic, and normal (WT: *n* = 4, *Mpc1^CKO^*: *n* = 4) tissue (**F**–**H**). *P < 0.05, **P < 0.01, ***P < 0.001. Values are represented as mean±SEM.

To understand the underlying mechanisms associated with the differential response to I/R injury in the *Mpc1^CKO^* and WT mice, we performed quantitative transcriptomics on hearts. Both groups were subjected to I/R, and, following 2 hours of reperfusion, we separated the heart into ischemic tissue (periinfarct area), and nonischemic tissue (away from the infarct) before RNA isolation and sequencing (Figure 2A). To identify differentially expressed genes, we used a 5% FDR with DeSeq2 software (version 1.24.0), and genes were filtered using an adjusted *P* value < 0.05, and absolute log_2_ fold change > 0.585. We performed ingenuity pathway analysis (IPA) as an integrative approach to identify enriched groups of genes among those that were differentially expressed.

To control for transcriptional changes that arose due to MPC deletion, we also obtained RNA from healthy WT and *Mpc1^CKO^* hearts at 4 WPI, before *Mpc1^CKO^* hearts show any cardiovascular phenotype. As expected, I/R injury led to large changes in gene expression (Supplemental Figure 1; supplemental material available online with this article; https://doi.org/10.1172/jci.insight.180906DS1). In response to I/R injury, there were 1,820 differentially expressed genes with a significant change (*P* < 0.05) in the *Mpc1^CKO^* hearts compared with WT hearts after multiple comparisons correction. Of these, 693 genes were differentially expressed in the ischemic myocardium, closest to the injury site (Figure 2B). Away from the myocardial injury, within the nonischemic myocardium, there were 1,122 genes differentially expressed in the *Mpc1^CKO^* heart compared with the WT heart(Supplemental Figure 2). Importantly, at this short time after tamoxifen treatment, there were almost no gene expression differences between WT and *Mpc1^CKO^* hearts.

When comparing the genes that are differentially expressed between ischemic WT and *Mpc1^CKO^* heart tissue, we noted that solute carrier family 16 member 3 (*Slc16a3:* red dot, black box), which encodes the lactate exporter MCT4, was the eighth most significantly upregulated gene in the ischemic myocardium of the *Mpc1^CKO^* tissue (Figure 2C). This is consistent with our hypothesis that lactate excretion provides an alternative route of pyruvate disposal to sustain glycolytic flux when mitochondrial import is blocked due to loss of the MPC. Additionally, we noted broader changes in the expression of genes associated with carbohydrate metabolism in response to I/R injury in the MPC1^CKO^ hearts (Figure 2D).

IPA analysis revealed an upregulation of several deleterious pathways in the *Mpc1^CKO^* ischemic myocardium compared with WT, such as interleukin signaling (*P* = 9.45 × 10^–14^), macrophage activation (*P* = 1.79 × 10^–11^), cytokine storm activity (*P* = 1.59 × 10^–10^), and cell death (*P* = 1.48 × 10^–05^), all of which may promote tissue injury following I/R (Figure 2E). We additionally examined gene signatures of cardiotoxic processes within *Mpc1^CKO^* hearts such as cardiac infarction (*P* = 2.13 × 10^–12^), fibrosis (*P* = 1.02 × 10^–11^), cell death (*P* = 4.80 × 10^–10^), and dysfunction (*P* = 1.61 × 10^–10^), compared with WT littermates (Supplemental Figure 3). In summary, following I/R, there is clear evidence of exacerbated tissue damage with a stress-associated transcriptional phenotype in *Mpc1^CKO^* hearts, including evidence of metabolic rewiring that includes upregulation of the *Slc16a3* gene.

### Mpc1^CKO^ hearts express high levels of MCT4 upon I/R.

We performed quantitative reverse transcriptase polymerase chain reaction (qRT-PCR) analysis to quantify *Slc16a3* and MPC1 expression in ischemic and nonischemic tissue of WT and *MPC1^CKO^* mouse hearts. Without I/R, *Slc16a3* gene expression was low and similar in both groups. Upon I/R injury, however, *Slc16a3* expression significantly increased in the WT ischemic and nonischemic heart tissue, which was exacerbated in the *Mpc1^CKO^* hearts (Figure 2F, qRT-PCR). To determine whether the *Slc16a3* gene expression pattern translated to increased protein abundance (Figure 2G), we performed immunoblotting and found that MCT4 protein abundance in *Mpc1^CKO^* mice was increased in the ischemic but not in the nonischemic myocardial tissue when compared with WT mice.

Interestingly, we note that the observed changes in MCT4 protein abundance after I/R are reciprocal to those of MPC1 (Figure 2H). MPC1 protein abundance within the ischemic area was significantly upregulated in WT mice, but, as expected, was much lower in the *Mpc1^CKO^* mice upon I/R. Our data show that at rest the myocardium lacking the MPC has low expression levels of *Slc16a3* (MCT4), similar to WT animals. Upon I/R injury, WT animals increase expression of MPC1 within the ischemic area, but in the *Mpc1^CKO^* heart MCT4 is induced.

### Mpc1^CKO^ cardiomyocytes under simulated I/R exhibit greater mitochondrial damage, increased cell death, and more lactate/succinate flux.

**Figure 3:**
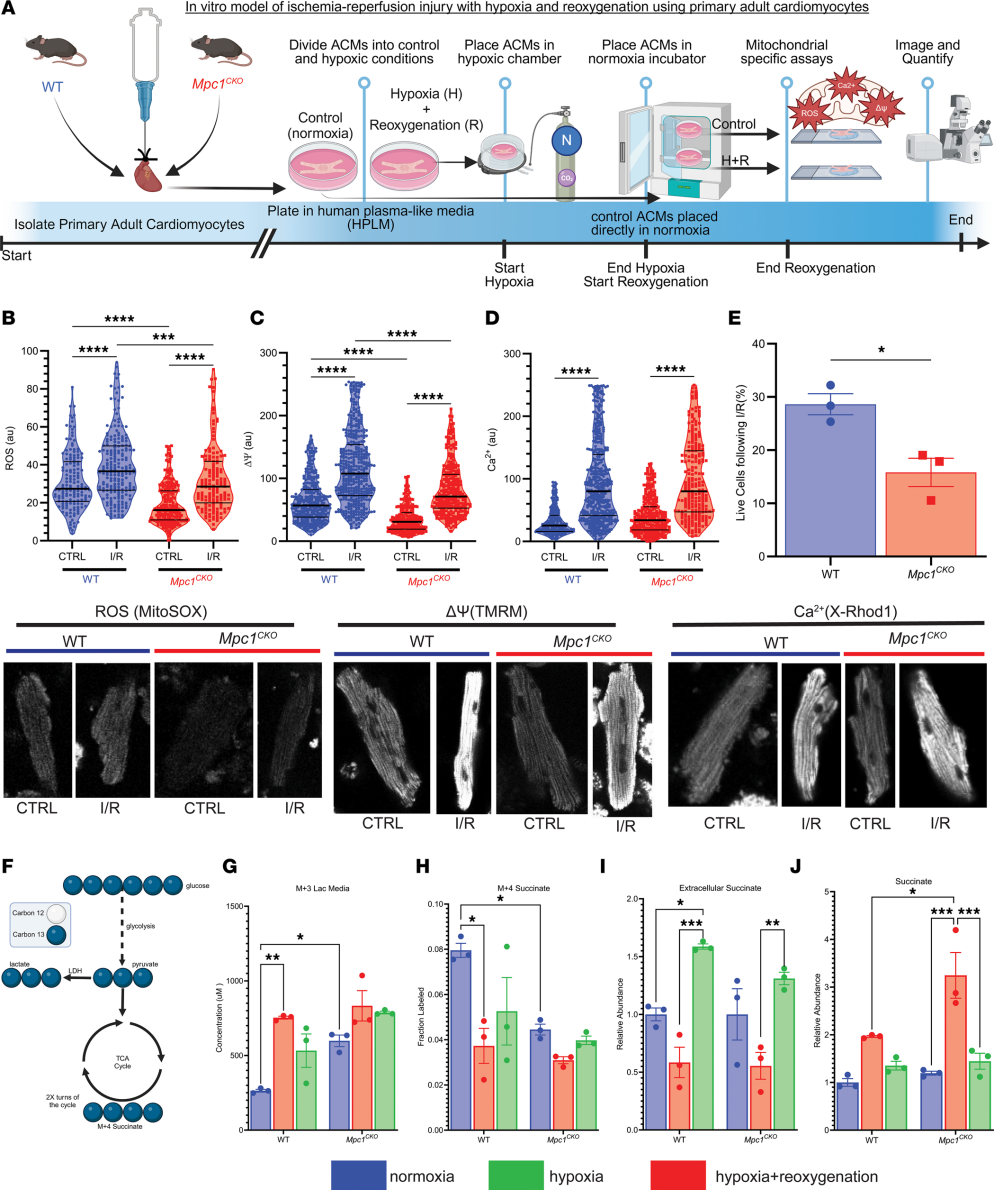
*Mpc1^CKO^* cardiomyocytes under simulated I/R exhibit greater mitochondrial damage, increased cell death, and more lactate/succinate flux. (**A**) Schematic workflow for in vitro adult cardiomyocyte ischemic/reperfusion (hypoxia + reoxygenation) injury. (**B**–**D)** Mitochondrial ROS, membrane potential; ΔΨ, Ca^2+^ following I/R showing increases from control cells (CTRL: baseline) to I/R, with greater changes in the *Mpc1^CKO^* cells. Magnification, ×40. (**E**) Percentage of live cells following I/R injury. (**F**) Diagram of [U-^13^C]glucose tracing in primary ACMs. (**G**) Media M+3 lactate from shows *Mpc1^CKO^* cells efflux more lactate from glucose into the media at baseline. (**H**) Cellular M + 4 succinate labeling from [U-^13^C]glucose–treated cells (**I**) Extracellular succinate measured during reperfusion. (**J**) Relative intracellular abundance of succinate during ischemia in WT and *Mpc1^CKO^*. Unpaired *t* tests (**E**) and 2-way ANOVAs with a Tukeys HSD post hoc test (**B**–**D** and **G**–**J**) were used for statistical analysis between WT (*n* = 3) and *Mpc1^CKO^* (*n* = 3), and normoxia, hypoxia, and hypoxia with reoxygenation. *P < 0.05, **P < 0.01, ***P < 0.001, ****P < 0.0001. Values are represented as mean±SEM. BioRender was used to make panels **A** and **F**.

To understand potential impairments in mitochondrial function in *Mpc1^CKO^* cardiomyocytes that may contribute to enhanced injury upon I/R, we utilized an in vitro model to simulate I/R. Following a previously described protocol, primary adult cardiomyocytes (ACMs) derived from 4 WPI WT and *Mpc1^CKO^* mice were isolated and exposed to 2 hours of hypoxia followed by 2 hours of reoxygenation (Figure 3A) (32). ACMs were then stained with mitochondria-specific reporters for ROS (MitoSOX), membrane potential (tetramethylrhodamine methyl ester, TMRM), or calcium (X-Rhod1) and imaged. *Mpc1^CKO^* ACMs cultured in normoxia showed less ROS and reduced ΔΨ compared with WT ACMs at baseline, potentially suggesting a lower energetic state, or even impaired mitochondrial fitness prior to I/R injury (Figure 3, B–D). Following simulated I/R injury, mitochondrial ROS, ΔΨ, and Ca^2+^ levels increased in both groups. Compared with WT cells, Mpc*1^CKO^* cells had a greater increase in ROS and ΔΨ, but not Ca^2+^ (Table 1). Consistent with our in vivo phenotype, simulated I/R injury resulted in significantly more cell death in *Mpc1^CKO^* cells compared with controls (Figure 3E).

We next performed uniform [U-^13^C]glucose tracing in ACMs to correlate changes in metabolism with mitochondrial function (Figure 3F). ACMs were exposed to hypoxia only, hypoxia and reoxygenation, or no treatment (normoxia, control). In WT ACMs, as expected, hypoxia induced a large increase in glycolytic flux as assessed by elevated media M+3 lactate excretion and this was partially reversed upon reoxygenation (Figure 3G). In contrast, in normoxia, *Mpc1^CKO^* cardiomyocytes efflux more M+3 lactate compared with WT ACMs and this was not significantly increased by hypoxia. Hypoxia suppressed M+4 succinate production in WT ACMs, but this was already depressed in *Mpc1^CKO^* ACMs and not further reduced by hypoxia (Figure 3H). Succinate is known to accumulate acutely in hypoxic cardiomyocytes and is associated with ROS injury upon reperfusion. Extracellular succinate accumulation was similar between genotypes in normoxia and decreased during hypoxia. Upon reoxygenation, similar amounts of succinate was exported extracellularly in both groups (Figure 3I). However, following hypoxia, the *Mpc1^CKO^* cardiomyocytes accumulated significantly higher levels of intracellular succinate compared with WT cells, which returned to near baseline levels in both groups after 2 hours of reoxygenation (Figure 3J). In summary, lactate secretion was increased and glucose oxidation decreased in *Mpc1^CKO^* cells at baseline, and these parameters were no longer responsive to changes in oxygen tension. Total intracellular succinate accumulated to a higher level in *Mpc1^CKO^* cells, raising the possibility that these cells are subject to greater metabolic reperfusion injury.

### Pharmacological inhibition of MCT4 attenuates I/R injury.

The observed upregulation of MCT4 accompanied by an increase in glycolytic flux leading to lactate excretion in WT hearts and ACMs subjected to I/R led us to investigate whether this metabolic adaptation was actually deleterious. We hypothesized that redirecting glucose carbon flux away from lactate secretion and toward oxidation would improve mitochondrial substrate availability and mitigate reperfusion damage. To test this, we employed a selective MCT4 inhibitor (VB124) at the time of reperfusion to block MCT4-mediated lactate export. By increasing intracellular lactate levels, the lactate-pyruvate equilibrium is altered, and through mass action, LDH produces more pyruvate that is shunted via the MPC toward mitochondrial oxidation. We previously demonstrated that MCT4 inhibition with VB124 prevented cardiomyocyte hypertrophy via a similar mechanism (18).

**Figure 4:**
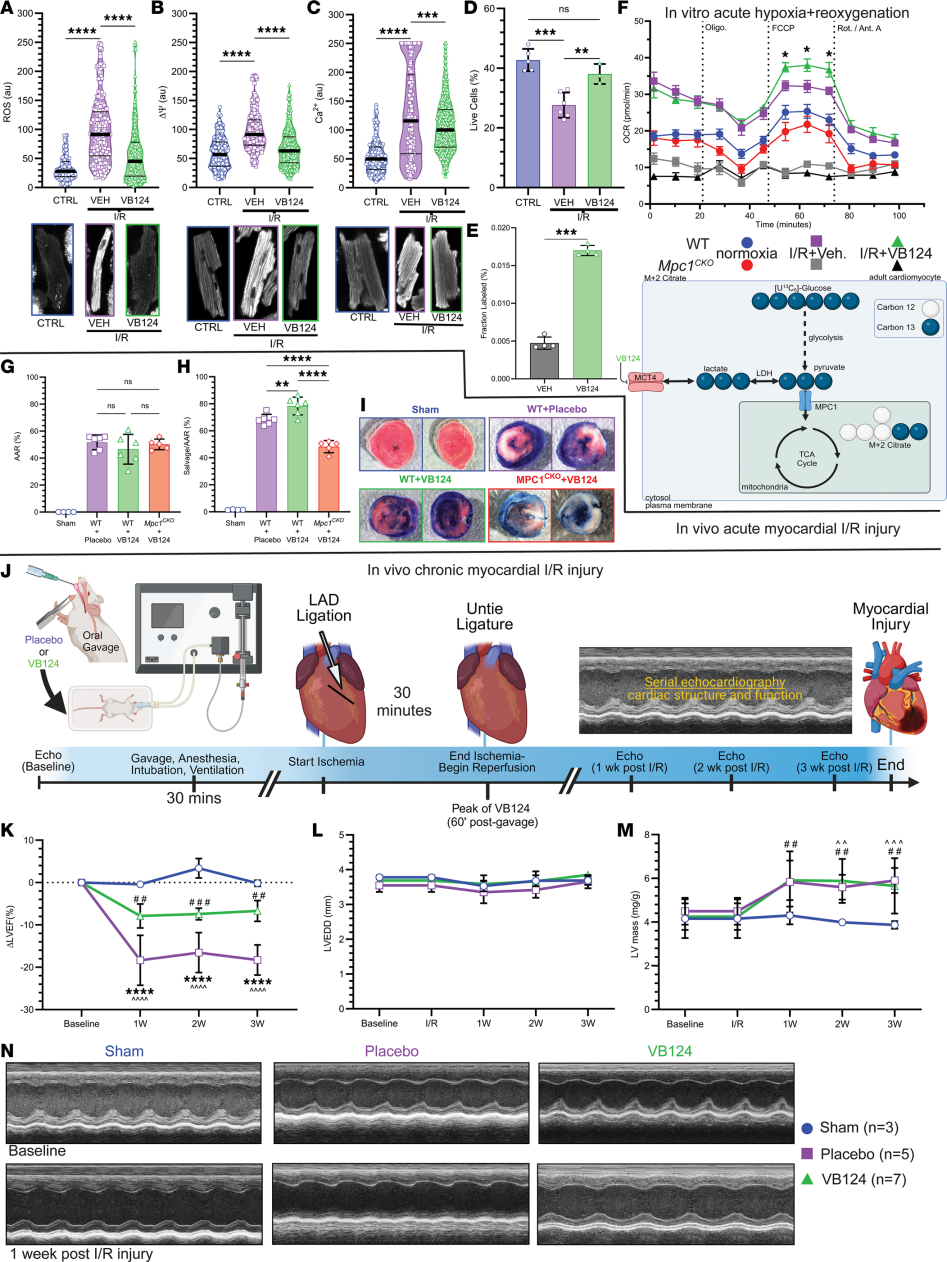
MCT4 inhibition alleviates I/R injury. (**A**) Mitochondrial ROS (**B**), membrane potential; ΔΨ (**C**), Ca^2+^ following hypoxia + reoxygenation with VB124 showing increases from baseline to I/R, and improvement with VB124 (normoxia: *n* = 5, –VB124: *n* = 6, +VB124: *n* = 3). (**D**) VB124 mitigates cell death following I/R injury. (**E**) M + 2 citrate labeling from [U-^13^C]glucose tracing in ACMs following hypoxia + reoxygenation with MCT4-inhibition (VB124) or Vehicle (DMSO) controls (*n* = 4, each). (**F**) Seahorse assay showing hypoxia + reoxygenation impairs *Mpc1^CKO^* mitochondrial respiration (normoxia: *n* = 6, vehicle: *n* = 7, VB124: *n* = 6) and VB124 (*n* = 9) improves maximal oxygen consumption rates in WT ACMs compared with Vehicle-treated WT ACMs (vehicle: *n* = 10, normoxia: *n* = 9). (**G**) Following I/R, the area at risk (AAR %) is nonsignificant, indicating similar injury between placebo, +VB124, and *Mpc1^CKO^* + VB124 groups (Sham: *n* = 4, Placebo: *n* = 6, VB124: *n* = 6, *Mpc1^CKO^* + VB124: *n* = 5). (**H**) Within the area at risk, myocardial salvage is significantly increased in *C57BL/6J* mice (*n* = 6), but not the *Mpc1^CKO^* (*n* = 5) gavaged with VB124, after I/R injury when compared with placebo (*n* = 6). (**I**) Representative images of myocardial salvage and necrosis following I/R injury in sham (*n* = 4), placebo (*n* = 6), +VB124 (*n* = 6), and *Mpc1^CKO^*+VB124 (*n* = 5) groups. Magnification, × 40(**J**) Schematic representation of in vivo chronic myocardial I/R injury with the placebo (methylcellulose) or VB124 oral gavage. (**K**–**N**) Following I/R injury, serial echocardiography was performed to assess cardiac function (left ventricular ejection fraction; LVEF) and structure (left ventricular end-diastolic diameter; LVEDD and left ventricular mass; LV mass). Unpaired *t* tests (**E** and **F**), and 1-way (**A**–**D**, **G**–**H**, and **K**–**M**) ANOVAs with a Tukey’s HSD post hoc test were used for statistical analysis. (**A**–**G**) **P < 0.01, ***P < 0.001, ****P < 0.0001. (**J**) ****P < 0.0001, placebo compared with VB124. (**L**) ^##^*P* < 0.01, VB124 compared with sham. ^^*P* < 0.01, placebo compared with sham. ^^^*P* < 0.001, placebo compared with sham. Values are represented as mean±SD. BioRender was used to make panels **E** and **J**.

Using our in vitro simulated I/R system, we treated ACMs from WT mice with VB124 at the time of reoxygenation. At baseline, there was minimal ROS emission and following hypoxia + reoxygenation, ROS increased. However, administration of VB124 at the time of reoxygenation blunted ROS levels following hypoxia + reoxygenation (Figure 4A). We witnessed a similar pattern on membrane potential, with low ΔΨ at baseline that was elevated following hypoxia + reoxygenation, but this increase was also mitigated by VB124 (Figure 4B). We observed the same pattern for Ca^2+^ (Figure 4C). We then measured the percentage of live cells (Figure 4D) in normal and hypoxia + reoxygenation injury conditions and observed enhanced cell death after hypoxia + reoxygenation that was rescued by VB124 administration. Next, we examined if MCT4 inhibition successfully increased pyruvate oxidation. Following hypoxia + reoxygenation, ACMs treated with VB124 and [U-^13^C]glucose showed a significantly higher fractional labeling of M + 2 citrate when compared to vehicle-treated control cells (Figure 4E) indicating an increased contribution from glucose carbon. Lastly, we measured oxygen consumption rates following hypoxia + reoxygenation via Seahorse assay (Figure 4F). Upon hypoxia + reoxygenation, *Mpc1^CKO^* ACMs were severely injured leading to reduced respiration rates following reoxygenation. However, WT ACMs that were treated with VB124 had improved maximal oxygen consumption rates after hypoxia + reoxygenation.

Encouraged by our in vitro results, we returned to an in vivo model of I/R injury and administered VB124 or placebo to healthy WT and *Mpc1^CKO^* mice (Supplemental Figure 4) via oral gavage. To determine the optimal timing for dosing, we orally gavaged mice with 30 mg/kg of VB124 and measured peak concentrations in the serum. VB124 reached a peak in the serum 1-hour after gavage and remained elevated for 4 hours (Supplemental Figure 5, left). Therefore, we timed myocardial reperfusion for 1-hour after gavage in sham, placebo, WT+VB124, and *Mpc1^CKO^*+VB124 mice. We collected serum and measured the concentration of VB124 by liquid chromatography-mass spectrometry (LC-MS) at baseline, at the end of ischemia, and at the end of reperfusion. This analysis showed that the serum concentration of VB124 was highest in the WT and *Mpc1^CKO^* mice prior to reperfusion of the myocardium, and nondetectable in the sham and placebo groups (Supplemental Figure 5, right). Controlling for infarct size, the AAR was similar between placebo, WT+VB124, and *Mpc1^CKO^*+VB124 mice (Figure 4G). The myocardial salvage was then normalized to the AAR, and WT mice gavaged with VB124 experienced a 15% increase in salvageable myocardium when compared with the placebo group, however, this cardioprotective response was abolished in the *Mpc1^CKO^* + VB124 mice (Figure 4, H and I).

Finally, we tested whether inhibiting MCT4 could enhance long-term recovery from I/R. To test this, we replicated our I/R protocol administering either a single dose of placebo or VB124 (30 mg/kg) via oral gavage prior to performing I/R. Unlike in the acute model, the mice were permitted to recover after surgery, and we performed serial echocardiography over a 3-week period to assess cardiac structure and function (Figure 4J). Initial assessments at baseline showed no significant differences between groups. Following 1, 2, and 3 weeks after I/R injury, left ventricular ejection fraction (LVEF) was significantly reduced in the placebo and VB124 I/R groups compared with sham-operated mice, however, this decrease was mitigated by VB124 treatment (Figure 4K). There were no significant changes to left ventricular end-diastolic diameter (LVEDD) between groups (Figure 4L). LV-mass increased similarly after injury in both the placebo and VB124 groups, and at 3 weeks after I/R, both groups had significantly larger LV-mass compared with the Sham group (Figure 4M). Sham-operated mice remained free of any signs of cardiac injury (Figure 4N). Our results show that acute MCT4 inhibition therapy suppresses increased mitochondrial ROS, ΔΨ, and Ca^2+^ following I/R injury, increased pyruvate flux to TCA cycle, and improved myocardial salvage leading to a sustained improvement in cardiac function.

## Discussion

Despite decades of research, no effective pharmacological therapies are available to treat myocardial I/R injury (2). Prior proposed interventions targeting metabolic aspects of I/R, such as protein kinase C inhibitors (30) and ATP-sensitive potassium channel openers (33), have not improved outcomes in the clinical setting, illustrating the need for new conceptual approaches. The MPC complex is responsible for transporting pyruvate, the end product of glycolysis, into the mitochondria for oxidation coupled to the synthesis of ATP. The MPC is upregulated at both the transcript and protein level in the surviving myocardium after I/R injury, suggesting what we believe to be a new mechanism of cardioprotection via enhanced pyruvate oxidation (22). Similarly, it was recently reported that the downregulation of *Mpc2* in the brain aggravates neuronal injury following cerebral I/R (34). Our group and others recently discovered that mice subjected to inducible heart-specific deletion of *Mpc1* spontaneously develop hypertrophic cardiomyopathy, become moribund, and perish 16 weeks after induction due to HF symptoms (18, 23–25). Based on the in vivo data showing a cardioprotective role of the MPC, we hypothesized that *Mpc1^CKO^* hearts would suffer from a worse I/R injury when exposed to a cardiac insult before the development of HF symptoms.

### Mpc1^CKO^ increases I/R injury.

We subjected *Mpc1^CKO^* mice 4 WPI with tamoxifen to acute I/R injury. Knockout animals suffered worse infarcts with 20% less myocardial salvage and more necrosis compared with littermate controls (Figure 1). Within the myocardium of *Mpc1^CKO^* animals, we observed an upregulation of several deleterious pathways and transcripts in the *Mpc1^CKO^* ischemic myocardium after I/R injury (Figure 2E). We also observed increased expression of *Slc16a3* (MCT4) (Figure 2F and 2G). MCT4 facilitates lactate efflux from cardiomyocytes, and its increased expression suggests that, in the absence of the MPC, pyruvate is being converted to lactate within the cytoplasm of these cells and exported. Of note, we also observed an injurious transcriptional phenotype within the nonischemic myocardium of the *Mpc1^CKO^* hearts, which indicated that the entire heart may undergo transcriptional changes following acute I/R injury.

Turning to an in vitro model to better characterize the cellular mechanisms underlying the genotype-specific responses to I/R injury, we exposed isolated ACMs to hypoxia before restoring normoxia and quantified ROS, ΔΨ, and Ca^2+^ as well as glucose metabolism using stable isotope tracers. *Mpc1^CKO^* ACMs had reduced baseline levels of ROS and ΔΨ compared with WT, suggesting that *Mpc1^CKO^* mitochondria may already exhibit an impaired energetic state. We can’t rule out that this baseline difference was exacerbated by the media conditions ACMs are cultured in, as these mice show no cardiac phenotype at this time point. The impaired fitness of *Mpc1^CKO^* mitochondria proved to be pathological as they demonstrated significantly more cell death with greater increases from baseline in response to stress in ROS and ΔΨ, but not Ca^2+^ (Figure 3 and Table 1). [U-^13^C]glucose labeling experiments demonstrated that, even in normoxia, *Mpc1^CKO^* have increased lactate excretion consistent with impaired pyruvate oxidation. During hypoxia, *Mpc1^CKO^* ACMs accumulated higher levels of succinate compared with WT cells, indicating impaired succinate oxidation/excretion following I/R injury, which has been shown to be detrimental (12). Our findings that cells with absent pyruvate oxidation show impaired mitochondrial responses to I/R injury strongly implicate pyruvate as a critical metabolite in conditions of hypoxia.

### MCT4 inhibition can alleviate I/R injury.

Pyruvate exists in an equilibrium with lactate catalyzed by the enzyme LDH. Lactate efflux from the cell is accomplished by the primary lactate exporter MCT4. Although MCT4 typically exhibits low expression in cardiomyocytes, it may be upregulated during pathological/stressful conditions that increase glucose consumption to fuel glycolytic metabolism in oxygen-limiting conditions such as hypoxia or extreme exercise (35, 36). While increased MCT4 may be an acutely adaptive response to heart injury, enhancing lactate efflux may actually be counterproductive as it facilitates increased LDH reaction flux, thereby siphoning pyruvate away from oxidation and enabling higher rates of glycolytic flux through rapid regeneration of cytoplasmic NAD+. In support of this idea, MCT4 inhibition therapy in mouse models has been established to prevent cardiomyocyte hypertrophy, and to attenuate cardiac injury following pulmonary embolism and chronic pressure overload (18, 37, 38).

Upon I/R, we observed enhanced expression of MCT4 in *Mpc1^CKO^* injured hearts compared with WT expression, likely due to high levels of intracellular lactate production. Returning to our in vitro hypoxia + reoxygenation model, administration of the MCT4 inhibitor VB124 at the time of reoxygenation mitigated the harmful effects of I/R injury, increased pyruvate oxidation, improved mitochondrial function, and rescued cardiomyocyte death (Figure 4, A–F). Respiration rates in the *Mpc1^CKO^* ACMs were nearly abolished following hypoxia + reoxygenation, despite VB124 treatment and a full serum complement of nutrients in the form of HPLM. This suggests that in the early stages of reperfusion cardiomyocytes are predominantly dependent on pyruvate oxidation for mitochondrial respiration.

We translated these findings in vivo in our acute I/R mouse model. We showed that VB124 treatment during I/R resulted in a 15% increase in myocardial salvage following injury when compared to placebo-treated mice (Figure 4, G–I). Interestingly, *Mpc1^CKO^* mice gavaged with VB124 did not show improvements to myocardial salvage following I/R injury, suggesting that MPC-mediated pyruvate transport is necessary for the cardiac benefit of MCT4 inhibition. Finally, we sought to understand whether these improvements in acute markers of heart injury would lead to long-term increases in cardiac function. Single-dose treatment with VB124 at the time of injury conferred durable cardioprotection, as shown by the preservation of cardiac function (LVEF) when compared with placebo-treated mice at 3 weeks after injury (Figure 4K). Interestingly, mice treated with either placebo or VB124 showed similar increases in normalized LV mass over body weight (mg/g), indicating that the hypertrophic response was similar between groups but functional consequences (differences in LVEF) perhaps reflected a reduced initial injury (Figure 4M).

These results show that MCT4 inhibition can be cardioprotective following I/R injury. Our data suggest that inhibiting MCT4 has the potential to alleviate acute and chronic I/R injury by redirecting the fate of pyruvate and improving mitochondrial oxidative metabolism. We hypothesize this may in part be due to the ability for MCT4 inhibition to increase mitochondrial pyruvate metabolism toward ATP production and away from other potentially deleterious metabolic pathways. Moreover, pyruvate has been shown to neutralize ROS by scavenging free radical species (39–41). This additional mechanism may protect the myocardium from oxidative stress and support the transition toward healthy cardiac metabolism following injury, however, more research is needed to support this hypothesis.

### Conclusions and perspectives.

Despite decades of research, there are currently no effective pharmacological therapies in clinical use today that alleviate I/R injury to the heart. Here, we present data to support pyruvate oxidation and the MPC to be cardioprotective in the setting of myocardial I/R injury. Our data in multiple systems show that the use of an MCT4 inhibitor could improve outcomes after I/R injury. Therefore, we suggest that interventions that promote pyruvate oxidation could alleviate I/R injury. Future research should focus on the exact mechanisms of how MCT4 inhibition can salvage the myocardium and address the efficacy, dose-response, and therapeutic window of this potential.

### Limitations.

In vitro, simulated I/R systems with hypoxia and reoxygenation are commonly used to study the mechanisms associated with I/R injury. However, the in vitro system has limitations that need to be taken into consideration. Chiefly, in vitro ACM systems lack mechanical stress and the complexity of diverse cell types, which may influence results. Moreover, the variability in the quality and health of primary ACMs during isolation and culture can introduce variation in results.

In vivo, the left anterior descending (LAD) artery occlusion is a widely used method to study I/R injury. In this study, the invasiveness of the in vivo I/R procedure requires surgery and can be associated with a risk of complications such as bleeding and mortality. For our tissue staining we were able to control for infarct size, however, the size of the infarct can vary depending upon the location of the occlusion and the extent of collateral branches that may be present. Additionally, the 30-minute duration of ischemia that we used may not accurately translate to longer periods of ischemia that may occur in patients.

## Methods

*Sex as a biological variable*. Our study examined male and female animals, and similar findings are reported for both sexes.

*Resource availability*. The key resources for this manuscript can be seen in Table 2.

### Experimental model and details

#### Animals and animal care.

To generate adult cardiac-specific *Mpc1*-KO mice, a C57BL/6J *Mpc1^fl/fl^* mice were bred to α*Mhc-Mercremer* mice. The resulting *Mpc1^fl/+^;* α*Mjc-Mercremer* were then bred to each other, resulting in the production of *Mpc1^fl/fl^;* α*Mhc-Mercremer* as well as their WT littermates (*Mpc1^fl/fl^-*α*mhc-cre^–/–^*), which were used as controls. To achieve adult cardiac-specific *Mpc1*-KO and WT littermates, 8-week-old *Mpc1^fl/fl^;* α*Mhc-Mercremer* and their WT littermates were intraperitoneally injected with 40 mg/kg of tamoxifen for 3 consecutive days.

#### In vivo I/R injury.

The experimental protocol was performed using 12–14-week-old mice (4 weeks after tamoxifen injection) prior to the development of HF symptoms, weighing 20–30 g and their WT littermate controls. At least 2 days prior to the scheduled surgery date, special attention was given to guarantee the cleanliness and the maximum sterility of the operation area and the well being of the mice. Consequently, clean surgical gown and sterile gloves were worn. The cleanness of the surgery area was checked and assured by bleaching the sterile area utilized as surgical zone and by using sterile drapes to delimit it. Finally, all surgery instruments were autoclaved and sterilized.

On the surgery day, mice were anesthetized with 2%–3% isoflurane inhalation, orally intubated with a 22 GIV catheter, and artificially ventilated with a small rodent respirator (Harvard Instrument). The level of anesthesia was monitored by testing for corneal reflex and muscle tone. The animals were ventilated at a rate of 100 breaths/minute with tidal volume adjusted to produce peak pressures of 15–20 mmHg. Anesthetized mice were placed on a circulating warming pad (38°C) in the supine position. Chest hairs were removed with a topical depilatory agent. Depilatory cream was then rinsed thoroughly with water or saline prior to surgical scrub to prevent skin irritation. The skin was then prepared with betadine and alcohol. A thoracotomy was performed to visualize the anterior surface of the heart. Next, a suitable retractor was used to retract the thoracic wall to improve visualization and accessibility. The pericardium was removed with the use of forceps and scissors. Under the dissecting microscope the LAD was visualized, and a 6-0 silk suture and the needle were inserted under the artery, as the needle entered the myocardium we were careful to avoid bleeding. A loose double knot was then made with the suture, leaving a 2–3 mm diameter loop through, then a 2–3 mm long piece of PE-10 tubing was placed. The tubing was soaked in 100% ethanol for 24 hours and rinsed free of alcohol with sterile water before use. The loop around the artery and tubing was tightened for 30 minutes to ensure coronary artery occlusion. The characteristic paler color appearance of the anterior wall of the LV appeared within a few seconds following LAD ligation, which we used as confirmation that the vessel was occluded. After the 30 minutes of ischemia was complete the knot was untied and the PE-10 tubing removed. The reperfusion was confirmed by observing a return of red color to the anterior wall of the LV after a few seconds. Then, the reperfusion continued for 120 minutes (2 hours). After completion of I/R injury, the coronary artery was briefly reoccluded, and Evans blue was injected into the left atrium to demarcate the AAR. Hearts were then excised, perfused with 0.9% saline, weighed, and then cut into 1-mm-thick transverse slices using a vibratome device, or separated into ischemic and nonischemic tissue samples to be flash frozen in liquid nitrogen. The slices were then incubated in 1% TTC in sodium phosphate buffer (pH 7.4) at 38°C for 20 minutes. TTC was used to stain the noninfarcted myocardium. The slices were then immersed in 10% formalin to enhance the contrast between stained (viable) and unstained (necrotic) tissue. Tissue sections were imaged the following day and myocardial salvage was quantified by independent researchers who were blinded to the treatment groups using ImageJ (NIH Software).

#### Echocardiography.

Under 1%–3% isoflurane anesthesia (VetOne Fluriso, NDC 13985-528-60), mice underwent echocardiography using the Vevo imaging system. Serial echocardiograms included 2D long-axis and short-axis views for analysis with Vevo strain software (version 3.1.1). Analysis was based on 2 consecutive cardiac cycles for all the measurements. Limb leads were used to record an ECG. To ensure objectivity, all echocardiography data were analyzed by an independent researcher who was blinded to the treatment groups.

#### Primary adult cardiomyocyte isolation and culture.

Adult mice were anesthetized and the heart was excised, attached to an aortic cannula, and perfused with solutions held at 37°C, pH 7.3. Perfusion with a 0 mM Ca^2+^ solution for 5 minutes was followed by 15 minutes of perfusion with the same solution containing 1 mg/mL collagenase and 0.1 mg/mL protease. The heart was then perfused for 1 minute with a “stopping solution” (the same solution containing 20% serum and 0.2 mM CaCl_2_). All perfusions were performed at a flow rate of 2 mL/min. The atria were removed, and the ventricles minced and filtered through a nylon mesh. Cells were stored at 37°C in culture media. All cardiomyocytes used in this study were rod shaped, had well defined striations, and did not contract spontaneously.

Following isolation, primary ACMs were enriched by gravity sedimentation in increasing concentrations of Ca^2+^ plating media. Cells were plated on Poly L-lysine coated Petri dishes and allowed to adhere for at least 1 hour in the incubator. Cells were then cultured in a final Ca^2+^ concentration of 1 mM.

#### In vitro I/R injury (hypoxia/reoxygenation).

Primary ACMs were isolated using the method described above and then placed in an airtight container with a constant gas flow of 95% nitrogen and 5% carbon dioxide at 37°C. This process ensured the complete removal of oxygen from within the sealed ischemia chamber. For ROS, ΔΨ, and Ca^2+^ measurements, the ischemia lasted for 2 hours. Following ischemia, cells were removed from the sealed container and placed in the oxygenated incubator alongside the control cells; measurements were taken following 2 hours of reperfusion. Control cells were kept at 37°C for the same amount of time that the experimental cells were exposed to the I/R conditions.

#### Tissue lysates.

Proteins from mouse heart were extracted from either ischemic, or nonischemic tissue samples. Briefly, tissue was placed in a 2 mL Eppendorf tube with 1 × RIPA lysis buffer, which contained HALT protease and phosphatase inhibitor cocktail solution (Pierce). A bead homogenizer (Tissue Lyser II, Qiagen) was used to homogenize the tissue samples and the lysates were incubated on ice for 1 hour. Samples were then centrifuged at 14,000*g* for 30 minutes at 4°C. Next, the supernatant was collected and the Pierce BCA Protein Assay Kit (Thermo Fisher Scientific) was used for protein estimation, before equal volumes of 2 × sample buffer containing 1 mM DTT was added to the samples.

#### Western blotting.

Following estimation of protein concentration, samples were boiled for 5 minutes at 95°C. 30–50 μg of total protein lysate was resolved on SDS polyacrylamide gel according to the standard procedure of 20 mA per gel and blotted onto a nitrocellulose membrane 0.45 μm (GE Healthcare) via Mini Trans-blot module (Bio-Rad) at a constant voltage of 100 V for 2 hours. Next, membranes were blocked with 5% proteomic grade nonfat dried milk (NFDM) for 1 hour and then the membranes were incubated overnight in 5% BSA. Antibodies probing for MPC1 (14462S Cell Signaling 1:1,000), and MCT4 (sc-376140 Santa Cruz 1:1,000) with a loading control of either Vinculin (13901S Cell Signaling 1:1,000) or HSP60 (12165S Cell Signaling 1:1,000) were used. Following incubation and on the next day, membranes were washed with TBS-T and placed in an incubation with a fluorophore conjugated secondary antibody (Rockland Immunochemical, 1:10,000) in 1% NFDM with TBS-T for 1 hour. Next, membranes were washed again with TBS-T and fluorescence was detected using an Odyssey CLx imaging system (LI-COR Biosciences).

#### Gene expression analysis.

Total RNA from mouse hearts (normal, ischemic, or nonischemic) was isolated using RNeasy Mini kits (Qiagen), according to the manufacturer’s instructions. Next, cDNA was synthesized using a cDNA Reverse Transcriptase Kit (New England Biolabs). TaqMan-based qRT-PCR were then performed using a QuantStudio 7 Pro Real-Time PCR System (Thermo Fisher Scientific). The housekeeping gene vinculin was used as an internal control for cDNA quantification and normalization of gene amplified products.

#### Mouse RNA-Seq.

Bulk RNA was isolated from mouse hearts (normal, ischemic, or nonischemic) using the miRNeasy Mini Kit (Qiagen). Total RNA samples (100–500 ng) were hybridized with Ribo-Zero Gold (Illumina) to deplete cytoplasmic and mitochondrial rRNA from the samples. Stranded RNA-Seq libraries were prepared as described using the Illumina TruSeq stranded Total RNA Library Prep Gold kit (20020598) with TruSeq RNA UD Indexes (20022371). Purified libraries were qualified on an Agilent Technologies 2200 TapeStation using a D1000 ScreenTape assay (cat# 5067-5582 and 5067-5583). The molarity of adaptor-modified molecules was defined by qRT-PCR using the Kapa Biosystems Kapa Library Quant Kit (cat# KK4824). Individual libraries were normalized to 1.30 nM in preparation for Illumina sequence analysis. Sequencing libraries (1.3 nM) were chemically denatured and applied to an Illumina NovaSeq flow cell using the NovaSeq XP chemistry workflow (20021664). Following transfer of the flowcell to an Illumina NovaSeq instrument, a 2 × 51 cycle paired end sequence run was performed using a NovaSeq S1 reagent kit (20027465).

#### RNA-Seq analysis and bioinformatics.

RNA-Seq analysis was conducted with the High-Throughput Genomics and Bioinformatics Analysis Shared Resource at Huntsman Cancer Institute at the University of Utah. Briefly, the mouse GRCm38 FASTA and GTF files were downloaded from Ensembl release 96 and the reference database was created using STAR version 2.7.0f with splice junctions optimized for 50 base-pair reads. Optical duplicates were removed from the paired end FASTQ files using BBMap’s Clumpify utility (v38.34) and reads were trimmed of adaptors using cutadapt 1.16. The trimmed reads were aligned to the reference database using STAR in 2-pass mode to output a BAM file sorted by coordinates. Mapped reads were assigned to annotated genes in the GTF file using featureCounts version 1.6.3. The output files from cutadapt, FastQC, Picard CollectRnaSeqMetrics, STAR, and featureCounts were summarized using MultiQC to check for any sample outliers. Differentially expressed genes with at least 95 read counts across all samples for a given tissue (normal, ischemic, or nonischemic) were identified using DESeq2 version 1.24.0. Genes were then filtered using the criteria FDR < 0.01, absolute log_2_ fold change > 0.0.585, (fold change > 1.516). Up and downregulated gene sets from each tissue were then analyzed for enriched GO terms using Gprofiler with a Benjamini-Hochberg FDR *P* value correction thresholded at < 0.01. RNA-Seq read counts were RPKM normalized for heatmap generation. Heat maps were generated by gene-standardizing the RPKM gene values (mean = 0, SD = 1) and plotting using XPRESSplot v0.2.2, Matplotlib, and Seaborn.

#### Metabolite extraction.

The protocols for metabolite extraction from cultured cells have been described previously. However, following the in vitro I/R injury (hypoxia/reoxygenation) the media was collected and aspirated. Adherent cells were washed with cold 0.9% sterile saline, which was chilled on ice. Next, 3 mL of extraction solvent (80% methanol/water) cooled to –80°C was added to the dish and then the dish was transferred to a –80°C freezer. Cells were then scraped into the extraction solvent on dry ice. All metabolite extracts were centrifuged at 20,000*g* at 4°C for 10 minutes. Each sample was then transferred to a new 1.5 mL tube. Lastly, the solvent in each sample was evaporated in a speed vacuum overnight and stored at –80°C until they were analyzed via mass spec.

#### [U-^13^C]glucose labeling.

Following cardiomyocyte isolation, the cells were placed in 10 cm plates with media where the normal glucose was replaced with ^13^C_6_-L-glucose (Cambridge Isotope Laboratories). Cells were allowed to incubate in the ^13^C_6_-L-glucose prior to the in vitro ischemic-reperfusion injury (hypoxia/reoxygenation) experiments. Following I/R injury, metabolites were extracted as described above and the data was corrected for naturally occurring ^13^C isotope abundance before analysis.

#### Seahorse mitochondrial stress test.

The seahorse mitochondrial stress test was performed following the manufacturer instructions on a Seahorse XF Pro Analyzer. The day of the experiment, ACMs were plated in 12 wells of a 96-well seahorse plate in HPLM media without BSA-conjugated fatty acids. The seahorse mitochondrial stress test was performed using 3 μM oligomycin, 1 μM FCCP and 1 μM rotenone/1 μM antimycin A, with a standard protocol consisting of 3 measurement cycles for each phase (2 minutes mixing, 3 minutes waiting and 3 minutes measuring). After the assay, data was analyzed in the Seahorse WAVE software through the XF Mito Stress Test Report.

### Statistics

For our statistical evaluations, we utilized GraphPad Prism software, version 10.0.0. The Shapiro-Wilk and D’Agostino-Pearson omnibus tests were used to verify the normality of data. ROUT testing was used to identify any significant outliers, and if identified were removed prior to analysis. We have presented data with either the mean *±* SD or ± SEM. We applied 2-tailed tests for all comparative analyses. When assessing 2 distinct groups, we employed the unpaired Student’s 2-tailed *t* test and the 2-tailed Mann-Whitney test. For evaluating serial echocardiographic data and multiple group comparisons, we conducted multiple 2-tailed *t* tests and 1- or 2-way ANOVAs, respectively. An α level of *P* < 0.05 was set a priori and considered significant. If necessary, a Tukey’s honest significant difference post hoc test was used for multiple comparisons.

### Study approval

All animal experiments conducted in this study were approved by and performed in accordance with the Institutional Animal Care and Use Committee (IACUC) at the University of Utah.

### Data availability

Data is available upon reasonable request to the corresponding authors. Mouse and other materials generated in this study are available for any researcher upon request. Transcriptomic RNA-Seq data has been deposited into NIH Sequence Read Archive (SRA) a public repository with NCBI (accession number: PRJNA1107578; https://www.ncbi.nlm.nih.gov/bioproject/PRJNA1107578/).

## Author contributions

JRV, AAC, JNVS, DC, SN, GSD, JR, and SGD were involved with the conceptualization of this manuscript. JRV, AAC, JNVS, DRE, LCR, DC, TSS, RH, JL, HK, and JYH were involved in methodology, validation, formal analysis, and investigation. DC and GSD contributed resources. IA, JRV, AAC, JNVS, LCR, and DRE were involved in data curation. Writing the original draft was completed by JRV, AAC, JNVS, GSD, SGD, and JR. Writing, reviewing & editing was completed by JRV, AAC, JNVS, GSD, JR, SGD, and SN. JRV, AAC, JNVS, DRE, LCR, TSS, RH, HK, and JYH contributed visualization. GSD, JR, and SGD supervised the project. Funding was acquired by JR and SGD. The order of the cofirst authors was assigned by a mutual agreement between JRV, AAC, and JV.

## Supplementary Material

Supplemental data

Supporting data values

## Figures and Tables

**Table 2:**
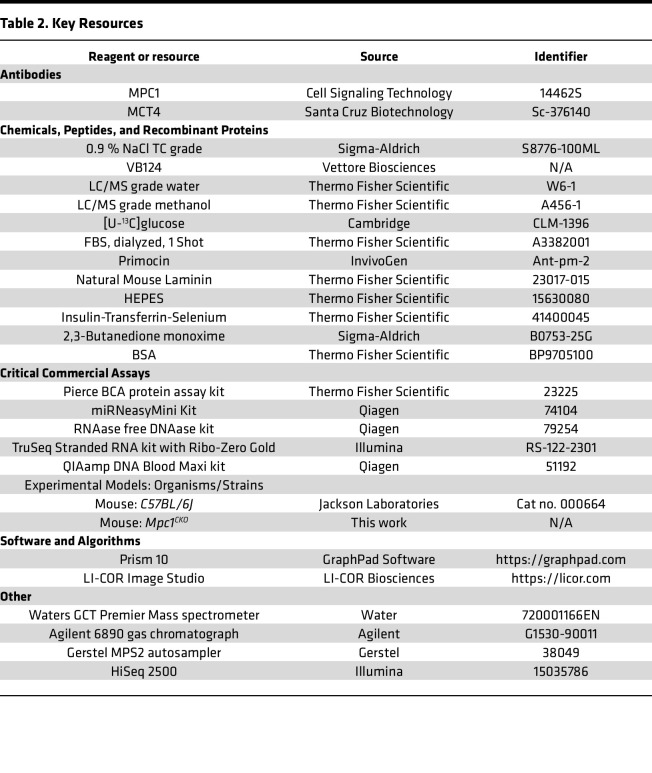
Key Resources

**Table 1:**
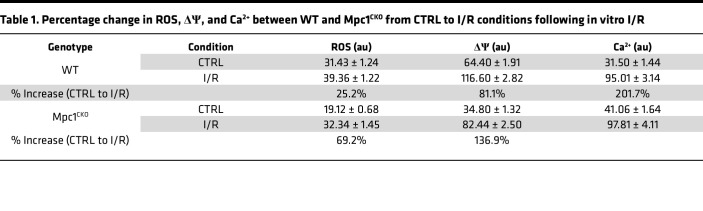
Percentage change in ROS, ΔΨ, and Ca^2+^ between WT and Mpc1^CKO^ from CTRL to I/R conditions following in vitro I/R

## Version 1 07/25/2024

In-Press Preview

## Version 2 09/10/2024

Electronic publication
