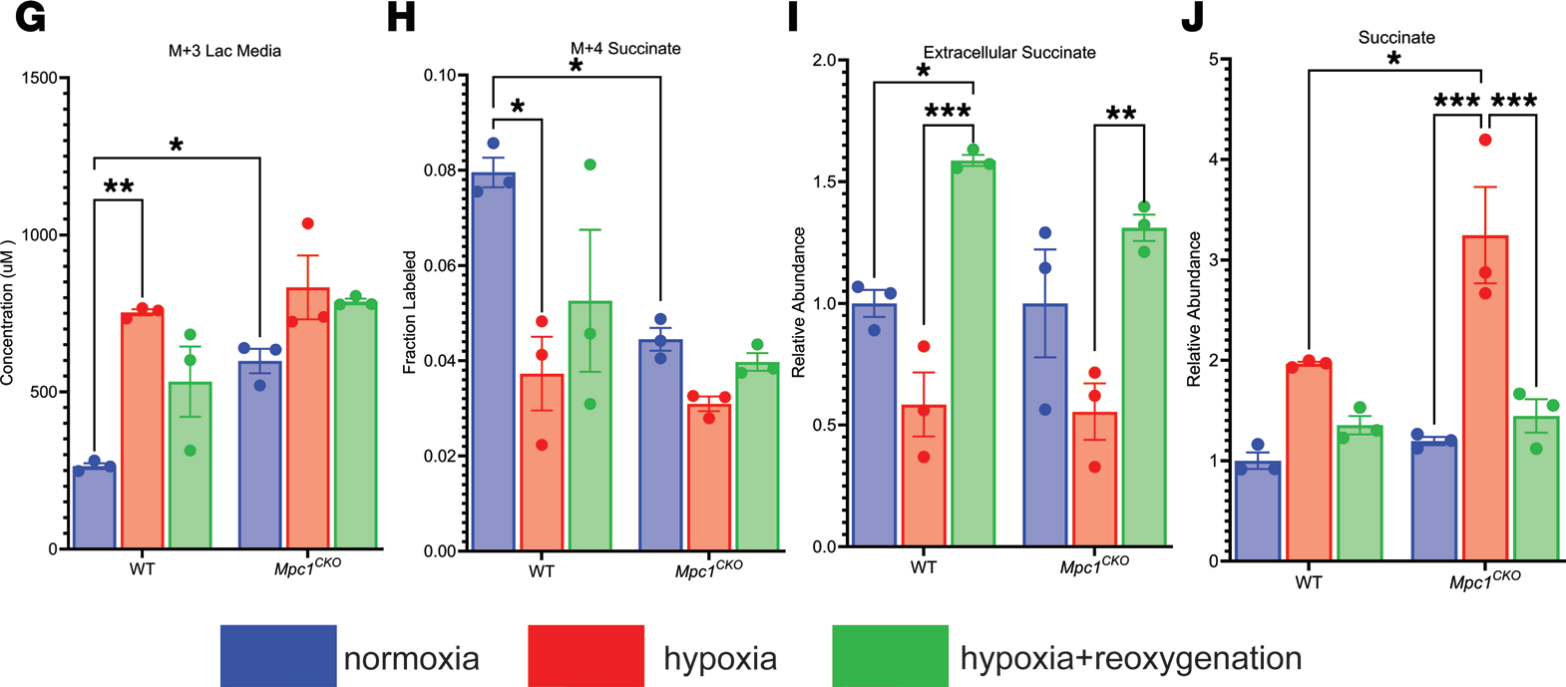# Enhancing mitochondrial pyruvate metabolism ameliorates ischemic reperfusion injury in the heart

**DOI:** 10.1172/jci.insight.187849

**Published:** 2024-11-08

**Authors:** Joseph R. Visker, Ahmad A. Cluntun, Jesse N. Velasco-Silva, David R. Eberhardt, Luis Cedeño-Rosario, Thirupura S. Shankar, Rana Hamouche, Jing Ling, Hyoin Kwak, J. Yanni Hillas, Ian Aist, Eleni Tseliou, Sutip Navankasattusas, Dipayan Chaudhuri, Gregory S. Ducker, Stavros G. Drakos, Jared Rutter

Original citation *JCI Insight*. 2024;9(17):e180906. https://doi.org/10.1172/jci.insight.180906

Citation for this corrigendum: *JCI Insight*. 2024;9(21):e187849. https://doi.org/10.1172/jci.insight.187849

After publication, the authors became aware of labeling issues in the paper. d-glucose was inadvertently referred to as l-glucose in the Methods. In addition, the key for Figures 3, G–J, was incorrectly assigned green to the hypoxia group and red to the hypoxia+regeneration group. The correct version of these panels is shown below. These errors have been corrected in the PDF and HTML versions of the manuscript.

The authors regret the error.

## Figures and Tables

**Figure 3:**